# Contrast-enhanced ultrasound performed under urgent conditions. Indications, review of the technique, clinical examples and limitations

**DOI:** 10.1007/s13244-012-0209-5

**Published:** 2012-12-18

**Authors:** Demosthenes D. Cokkinos, Eleni Antypa, Ioannis Kalogeropoulos, Dimitrios Tomais, Emmanuel Ismailos, Ioannis Matsiras, Stylianos Benakis, Ploutarchos N. Piperopoulos

**Affiliations:** 1 Radiology Department, Evangelismos Hospital, 45-47 Ypsilantou Street, Athens, 10676 Greece; 2 CT-MR Department, Evangelismos Hospital, 45-47 Ypsilantou Street, Athens, 10676 Greece

**Keywords:** Ultrasound, Contrast-enhanced ultrasound, CEUS, Ultrasound contrast agents, Urgency

## Abstract

Contrast-enhanced ultrasound (CEUS) is an imaging technique with various indications, most of which refer to scheduled examinations. However, CEUS can also be performed under urgent conditions for the investigation of many different clinical questions. This article reviews basic physics of ultrasound contrast agents and examines the commonest urgent clinical applications of CEUS. These include, among others, abdominal solid organ trauma and infarcts, scrotal and penile pathology and blood vessel imaging. Patients can be examined with a very short time delay at their bedside, without exposure to ionising radiation or risk of anaphylactic reaction and renal failure, while contraindications are minimal. CEUS technique is described for various urgent indications and imaging examples from our department’s experience are presented. Safety matters and limitations of CEUS are also mentioned.

*Teaching Points*

- *Contrast-enhanced ultrasound (CEUS) can be performed urgently for various clinical applications.*
- *Abdominal indications include solid organ trauma and infarcts.*
- *CEUS in abdominal organ trauma correlates well with CT and can replace it for patient follow-up.*
- *CEUS images testicular torsion, infection and infarction, as well as testicular and penile trauma.*
- *Blood vessels can be assessed with CEUS for obstruction, aneurysm, thrombosis and dissection.*

## Introduction

Ultrasound (US) contrast agents have gained a significant role in clinical practice, being used in more than 50 countries [1]. Although most contrast enhanced ultrasound (CEUS) studies are scheduled in advance, patients may be examined urgently provided that they are haemodynamically stable. Urgency is a situation requiring fast action for diagnosis and treatment. For CEUS, these cases mainly include solid abdominal organ injuries and infarcts, vessel pathology, as well as scrotal and penile pain. CEUS examinations are performed if there is strong clinical suspicion of urgent pathology, even if appropriate findings are not seen on baseline, non-enhanced US examination carried out before contrast injection.

Although a delay in the US department is unwanted in haemodynamically unstable patients, stable patients can be imaged quickly without substantial time loss. If an intravenous cannula is already inserted, which usually happens in emergency departments, CEUS hold-up can be less than 10 min. US contrast agents are non-nephrotoxic, therefore not contraindicated in patients with renal insufficiency, while anaphylactoid reactions are practically non-existent.

This article refreshes basic knowledge of US contrast agents and explains the examination technique in an urgent setting. We describe indications, pitfalls and limitations and depict various clinical examples.

## Basic principles, physics and safety of CEUS

Ultrasound contrast agents are composed of gas-filled microbubbles with low solubility in blood [1], coated with a shell of different proteins, lipids or polymers [2]. Being too big to pass through the endothelial vessel wall, they are pure intravascular agents [3].

Air-filled, first-generation, high mechanical index (MI) contrast agents (e.g. SHU508, Levovist) are not administered for clinical studies anymore. Second-generation agents (e.g. BR-1, Sonovue) work in low MI conditions. When a US wave reaches the agent, they show asymmetric oscillation, producing echoes containing harmonic frequencies [4], which are signals with frequency peaks at multiples of the transmitted frequency. Second generation contrast agents stay intact for up to 7 min in low MI and are used in everyday clinical practice.

Contrast imaging requires dedicated contrast agent-specific software [5, 6] to improve contrast resolution and suppress stationary tissue signal [7], such as phase inversion: two US pulses of constant amplitude with a change of phase of alternate pulses by 180° [1] are sent sequentially. Returning signals are added up by the US machine [8], resulting in almost complete stationary tissue cancellation, with a strong contrast agent signal. A split screen simultaneous view of contrast enhancement next to the baseline grey scale image enables operator orientation in the baseline US reference image, while at the same time following tissue enhancement. In our hospital, we use SonoVue, a blood pool contrast agent, which consists of a stabilised aqueous suspension of sulphur hexafluoride microbubbles, coated by a phospholipid shell [9].

US contrast agents’ behaviour is generally similar to computed tomography (CT) and magnetic resonance (MR). Their main difference is that, since US agents are not excreted by the kidneys, they are not contraindicated in patients with poor renal function.

CEUS images blood flow better than colour and power Doppler, by overcoming the limitations of these techniques [10–12]. Although Doppler identifies blood with sufficiently fast flow compared with tissue movement, in the parenchyma of most organs blood may be moving at a very low speed, not easily discriminated from tissue motion [1]. US contrast agents detect parenchymal microvasculature in vessels with very small size and with too low a velocity that cannot be imaged by Doppler. Microbubbles are even detected if stationary. While in contrast enhanced CT and MR only still images can be obtained, microbubble uptake can be seen in real time for a time period of up to 5–7 min.

US contrast agents are very well tolerated with 1:7,000 (0.014 %) anaphylactoid reactions [1, 13, 14], rates lower than the equivalent of CT contrast agents (0.035–0.095 %) [1, 15, 16]. A previous United States Food and Drug Administration (FDA) warning when using certain US contrast agents in patients with severe cardiopulmonary compromise [17–19] was recently modified. Nevertheless, there is no current FDA-approved CEUS abdominal radiology indication in the United States [1]. Around the world, however, US contrast agents are used with practically no adverse reactions and are well tolerated [9].

## Clinical urgent applications, technique description and examples

### CEUS in abdominal trauma

Baseline non-enhanced US is the commonest imaging examination for blunt abdominal trauma [20–22], focusing on detecting intra-abdominal, pleural and pericardial fluid with sensitivity rates up to 99 % [23, 24] and is known as FAST (focused assessment with sonography for trauma) ultrasound [25]. Nevertheless, its value is limited for showing traumatic lesions of solid abdominal organs [26], since contusions may be isoechoic to hepatic, splenic or renal parenchyma [27], while about a third of solid organ injuries are not combined with haemoperitoneum [28, 29].

**Fig. 1:**
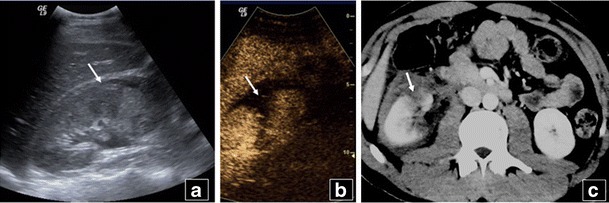
Follow-up US of a 25-year-old male patient with right kidney rupture, initially imaged with CT. There is loss of right kidney architecture and small fluid collection on B mode US (*arrow* in **a**). CEUS shows rupture confined to the anterior part of the kidney (*arrow*) and haematoma (**b**). The admission contrast enhanced CT (**c**) performed 6 days earlier had diagnosed right kidney rupture (*arrow*) affecting anterior and posterior parts of the kidney, while the fluid collection was larger. No additional CT was performed until the patient’s discharge

Conversely, although contrast-enhanced CT is the “gold standard” technique for abdominal trauma, often patients eventually have no or minor injuries, a scenario often seen in unilateral localised sports, playground and low-altitude fall injuries [30]. In these cases, CEUS can prove very useful, with sensitivities for detecting injuries of 69 % (kidneys), 84 % (liver) and 93 % (spleen) compared with CT and very high specificity (over 90 %) [31]. Thus, CEUS has developed a role in this urgent setting to evaluate low-energy injuries. This can be performed during initial evaluation but also during follow-up [32]. Even if patients have been initially scanned with CT, localised injuries can later on be followed with CEUS (Fig. 1), reducing CT scans, especially when imaging young patients [33].

**Fig. 2:**
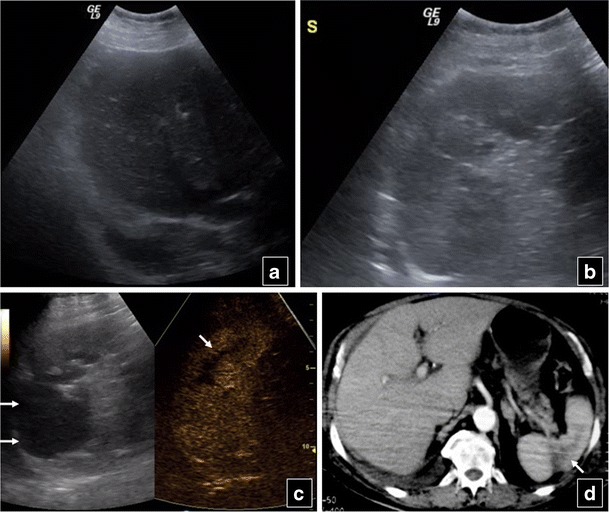
A 79-year-old woman who fell from a height. Baseline US shows perihepatic and right pleural effusion (**a**) as well as splenic inhomogeneity and small left pleural effusion (**b**). CEUS (right part of **c**) reveals a splenic contusion located in the middle and lower part, parallel to the organ’s axis (*arrow*). The upper part of the spleen, although appearing hypoechoic on baseline US (*double arrows*) is intact on CEUS, showing normal enhancement. Contrast-enhanced CT confirms splenic contusion, the shape and size of which correlate very well with CEUS (*arrow* in **d**). Perihepatic fluid is also noted

**Fig. 3:**
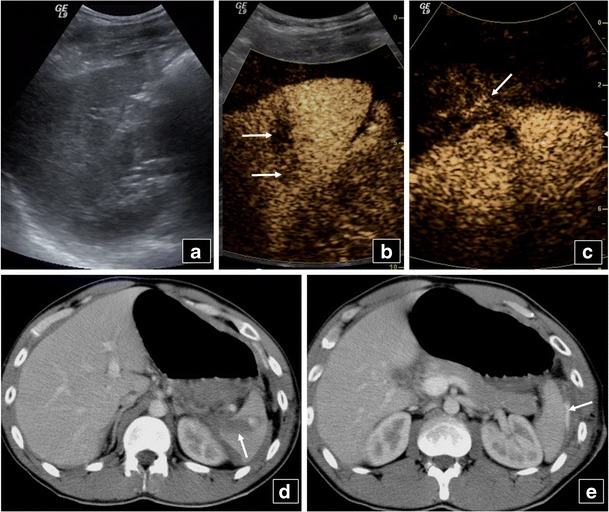
A 31-year-old man who fell from a low height. Baseline US (**a**) reveals only inhomogeneous echogenicity of the spleen. On CEUS a large contusion is clearly seen (*arrows* in **b**), along with contrast extravasation (*arrow* in **c**) due to ongoing haemorrhage. CT performed 1 h later confirmed splenic contusion (*arrow* in **d**) and extravasation (*arrow* in **e**)

On CEUS, solid organ injuries appear as non-enhancing areas [30, 34, 35] and haematomas show no internal enhancing vessels [34]. CEUS accurately defines organ injuries, capsular extension and even vascular injury [32] with very good correlation with CT (Fig. 2). Ongoing haemorrhage is seen as contrast extravasation pooling or jet outside blood vessels (Fig. 3) [34, 36, 37].

**Fig. 4:**
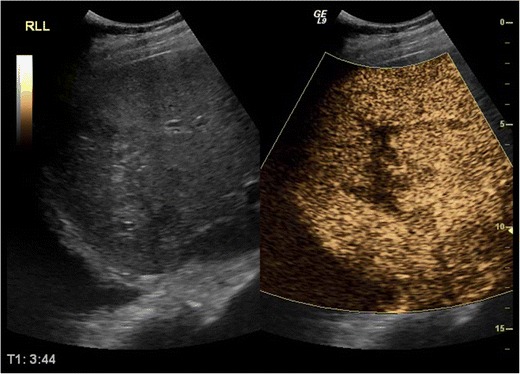
CEUS in a 44-year-old man shows an enhancing defect in the right lobe of the liver due to contusion (right part of image). Baseline US (left part of image) is not diagnostic of parenchymal injury, revealing only inhomogeneous hepatic echogenicity
  Besides trauma, hepatic infarcts are also often seen on baseline US. They appear as hypoechoic triangular areas with base facing lateral parts of the organ. In dubious cases with isoechoic or slightly hypoechoic lesions on unenhanced scanning, infarcts appear as enhancement defects on CEUS. Correlation with CT is very good, therefore the latter can be avoided if the examiner is adequately experienced in CEUS imaging (Fig. 5).

Patients examined with US for the evaluation of abdominal trauma are initially scanned using the FAST examination protocol [38, 39], followed by baseline unenhanced examination of the liver, spleen or kidney on the patient’s side of injury. If indications exist (localised pain, focal lesions of suspicious echogenicity or inhomogeneities more intense than different grades of fat deposit, abdominal fluid) an urgent CEUS examination can be carried out. Certain technical aspects need to be kept in mind while examining different organs:

- Liver
  The dose administered varies from 50 % to 100 % of the dose used for characterising focal liver lesions to avoid possible obscuring of thin lacerations. In our institution, we use 2.4 ml SonoVue for adults (Fig. 4). The dose for children is determined as follows: millilitres of SonoVue = age in years/10 [30, 32, 40]. Studying its dual blood supply from hepatic artery and portal vein, liver arterial phase imaging detects active bleeding, while late phase scanning better evaluates lacerations [32].

**Fig. 5:**
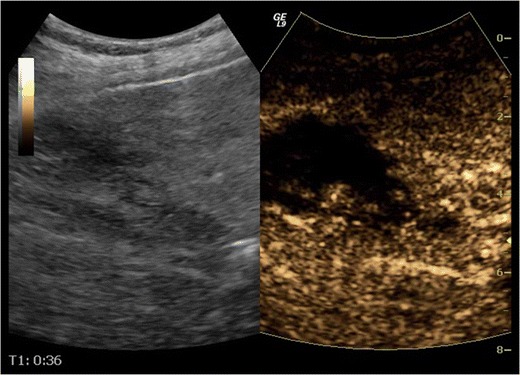
Hepatic infarct in a 67-year-old man with sickle cell disease. The triangular ill-defined hypoechoic lesion on baseline US (left part of figure) is better delineated on CEUS (right part)
- Spleen
  Post injection, the spleen shows a long arterial inhomogeneous enhancement caused by different perfusion rates between red and white pulp, which disappears around 60 s post injection. Thus, the delayed phase is important to minimise erroneous interpretation of early heterogeneous enhancement as injury [32]. The late phase lasts up to 7 min, allowing plenty of time to examine the whole organ. Moreover, the splenic vein and branches show late contrast washout, appearing as defects about 2–3 min post injection, probably because the spleen acts as a filter for the agent’s microbubbles [30]. Thus, the splenic vein (it has a tubular appearance with branching vessels and at least a few microbubbles in its lumen) should be differentiated from lacerations (their shape is irregular, with no branches or microbubbles). A shattered spleen presents an ill-defined outline and loss of normal architecture (Fig. 6). The doses suggested if SonoVue is used vary between 0.6 ml and 25–50 % of the respective adult hepatic dose. Paediatric dose is determined as follows: millilitres of SonoVue = age in years/20 [30, 40].

**Fig. 6:**
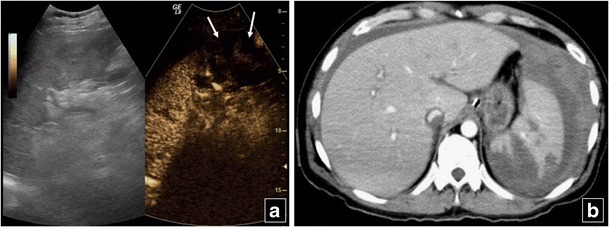
Splenic rupture in a 53 year old man. Baseline US (left part of **a**) shows inhomogeneous echogenicity in the lower part of the spleen. An ill defined perisplenic fluid collection with echogenic content is also suspected. CEUS (right part of **a**) reveals completely deranged echogenicity of the ruptured part of the spleen with no contrast uptake (*arrows*). The fluid collection is now clearly outlined from the spleen, obviously due to haemorrhage. Findings of splenic rupture and fluid collection are confirmed on CT (**b**)
- Kidneys
  The optimal time period for kidney CEUS assessment is up to 2.5 min [40]. Consequently, the time for CEUS kidney scanning is shorter compared with the liver and spleen. Injured areas appear as filling defects (Fig. 7), while haematomas are very well outlined on CEUS, appearing anechoic (Fig. 8). Infarcts can also be well imaged on CEUS as filling defects (Fig. 9). The dose used is the same with the splenic dose [30, 32, 40].

**Fig. 7:**
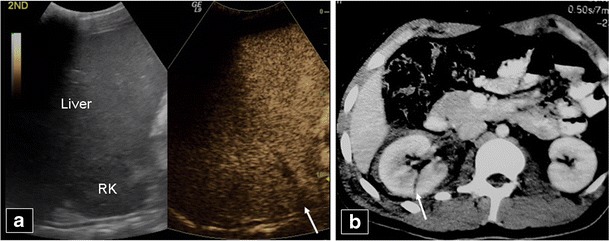
A 50-year-old man, who was a car accident victim, was followed-up with CEUS (**a**) after initial imaging with CT (**b**) 9 days earlier. CEUS shows a thin right kidney (*RK*) rupture (*arrow* in **a**) with no perinephric fluid. The initial CT scan had diagnosed the kidney rupture (*arrow* in **b**) with a collection which subsided by the time CEUS was performed. The patient did not undergo an additional CT scan

**Fig. 8:**
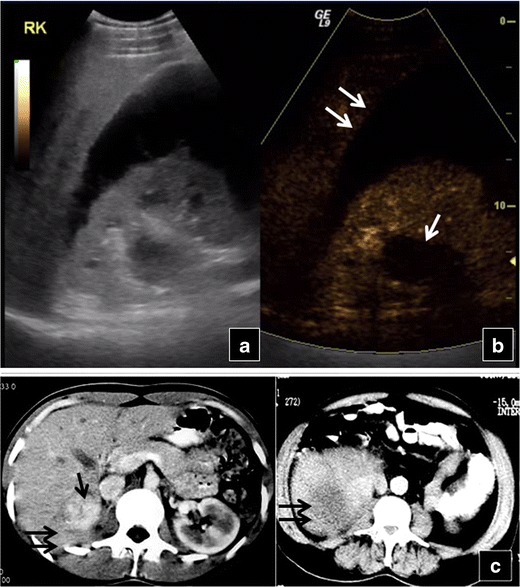
Baseline US (**a**) of a 25-year-old man reveals a hypoechoic area in the middle of the right kidney with a large perinephric collection. CEUS (**b**) elucidates the presence of renal rupture (*arrow*) and large haematoma (*double arrows*). CECT (**c**) confirms right kidney rupture (*arrow*) and haemorrhagic collection (*double arrows*)

**Fig. 9:**
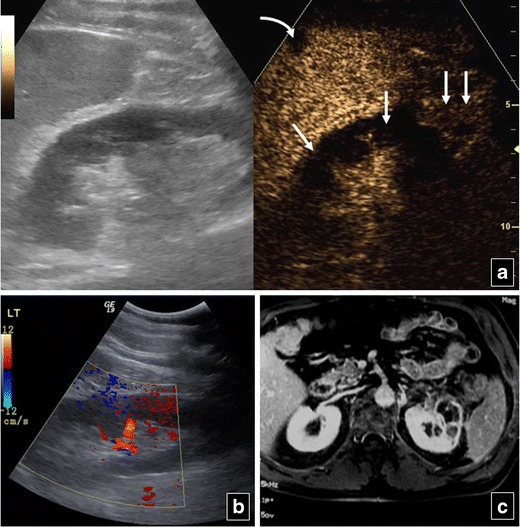
Inhomogeneous echogenicity is noted on B mode US (left part of **a**) of the left kidney in a 56-year-old man. Colour Doppler US (**b**) is suboptimal for the detection of blood flow in the renal cortex. CEUS (right part of **a**) shows absence of flow in parts of the cortex due to partial infarction (*arrows*), while other parts of the cortex (*double arrows*) and medulla enhance normally. A small splenic infarct (*curved arrow* in **a**) is also seen on CEUS. Left kidney partial cortical infarction is confirmed on MR (**c**). The splenic infarction is not seen at this level

#### Examining more than one abdominal organ for trauma

Although CEUS is mainly used to assess an isolated injury on one flank [30], it is possible to image more than one abdominal organ. In stable patients with a specific injury detected in the first 2–3 min post contrast injection, the remaining 2–5 min can be spent to scan additional organs, dividing a full dose into two or three smaller doses. The first dose is administered with the patient in the left decubitus position, dedicated to the right kidney, (first 2 min) and liver (remaining 3 min) [6, 30, 35]. The second dose is given in the right decubitus position, the first 2 min spent for the left kidney and the remaining time (up to 5 min) for the spleen.

#### CEUS in combination with CT for trauma

If no injuries or only limited unilateral abdominal injury are seen on CEUS, stable patients can be observed without an urgent CT, thus economising on ionising radiation, time and money. However, in patients with multiple injuries, CEUS should not replace contrast-enhanced CT, which remains the method of choice. Critical patients should not be assessed with CEUS but, immediately after FAST, should be transferred to CT (if stable) or to surgery (if unstable) [30, 40, 41]. If CT has resulted in uncertain findings due to its own pitfalls (possible suboptimal contrast uptake, artefacts due to arms, medical device superimposition or patient motion), CEUS can be used to further evaluate [34]. Altogether, although CEUS cannot replace CT, it may reduce its use as a screening method.

CEUS can also play a role in trauma follow-up. If patients have followed an uneventful hospitalisation period, they can be imaged subsequently with CEUS [34], leading to the reduction of repeat CT scans, especially in young ages [33]. CEUS can also be used to follow-up patients after embolisation for splenic trauma [42]. In our practice, patients with abdominal injuries on admission CT who are improving during their stay in the hospital are further assessed with CEUS, potentially until the resolution of their injury.

### CEUS in testicular and penile urgency

Ultrasound is the first imaging test performed to evaluate scrotal pathology, mainly comprising torsion, trauma, infection and tumours [43]. CEUS can confirm B mode and colour Doppler US findings, especially when these are equivocal, aid in differential diagnosis and rule out complications [44]. Although the same dose as for the liver may be used, in our experience, larger doses offer better enhancement (e.g. 4.8 ml for Sonovue).

#### Testicular torsion

**Fig. 10:**
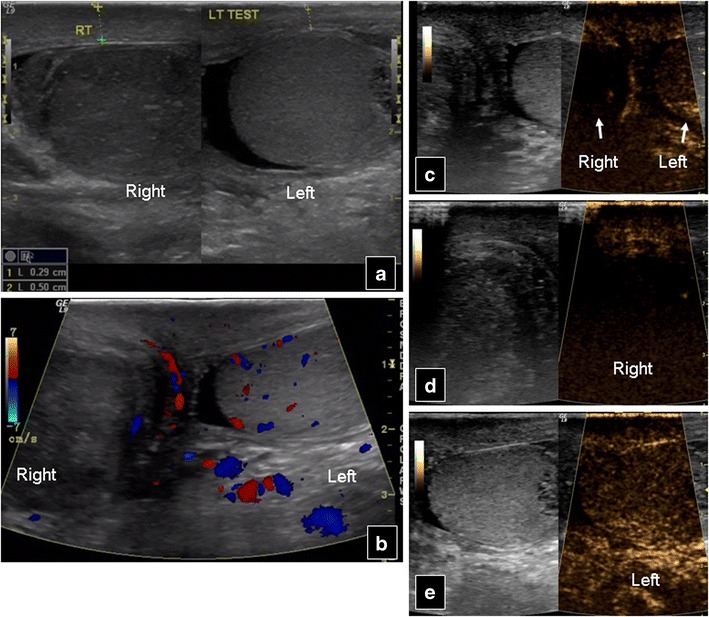
A 25-year-old patient with enlarged and painful right testicle. Baseline US (**a**) shows a large hypoechoic right testicle with scrotal wall thickening in comparison to the normal left side. Colour Doppler US (**b**) shows absence of vascularity in the right testicle. Post-contrast injection, torsion is confirmed with absence of enhancement in the right testicle in comparison to the left: (**c**) transverse view of both testicles, (**d**) sagittal view of the right testicle, (**e**) sagittal view of the left testicle

Usually colour and power Doppler US are sufficient to diagnose testicular torsion, with CEUS kept to confirm vascularity absence (Fig. 10) in difficult cases, such as in adolescents with small testes where colour Doppler information is suboptimal. Altogether, however, CEUS has not proved to add information in studying complete testicular torsion [44]. Nevertheless, in chronic (initially missed) torsion, CEUS can detect peritesticular increased vascularity, while in incomplete torsion it reveals enhancement discrepancy between normal and abnormal testicle. It can also help in intermittent torsion, with normal or increased flow. In such cases differentiation from orchitis [45] may be needed. It can be difficult to differentiate a segmental infarct with a rounded configuration from a poorly vascularised tumour [46, 47]. CEUS can help in this task, allowing a more confident diagnosis, compared with grey-scale and colour Doppler [48].

#### Testicular and penile trauma

**Fig. 11:**
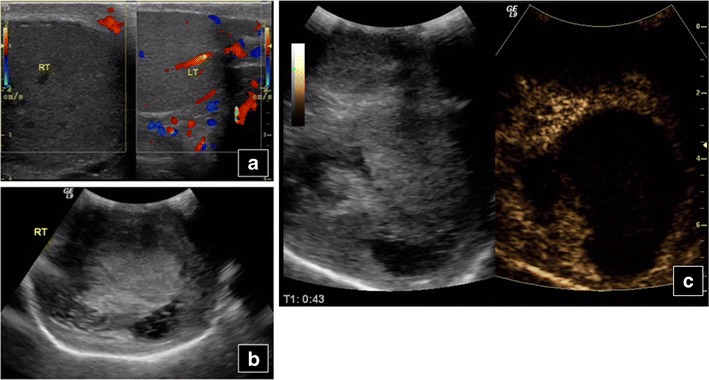
Testicular trauma in a 42-year-old man. Colour Doppler US (**a**) shows enlargement with no flow in the right (*RT*) injured testicle in comparison to the normal left (*LT*). B mode US (**b**) reveals loss of architecture on the right side. Complete absence of perfusion in the right testicle is confirmed on CEUS (**c**)

US shows testicle border alteration, haematoma, haemorrhage or infarction with altered echogenicity, thickened testicular or scrotal wall and tunica albuginea loss of continuity. Injuries appear as hypoechoic unenhancing focal areas. Ruptured testes show loss of normal architecture and lack of enhancement [44]. Again, CEUS confirms colour Doppler US findings (Fig. 11), assessing trauma degree, potentially underestimated on unenhanced US [34]. Haematoceles and haematomas dο not enhance. If CEUS can detect blood flow, an actively bleeding haematoma or neoplasm should be suspected. CEUS improves colour Doppler assessment, by defining fracture lines, haematomas and viable tissue amount [48]. As 10–15 % of tumours are seen after trauma [49], testicular abnormalities detected post traumatically should be sonographically followed until they resolve.

**Fig. 12:**
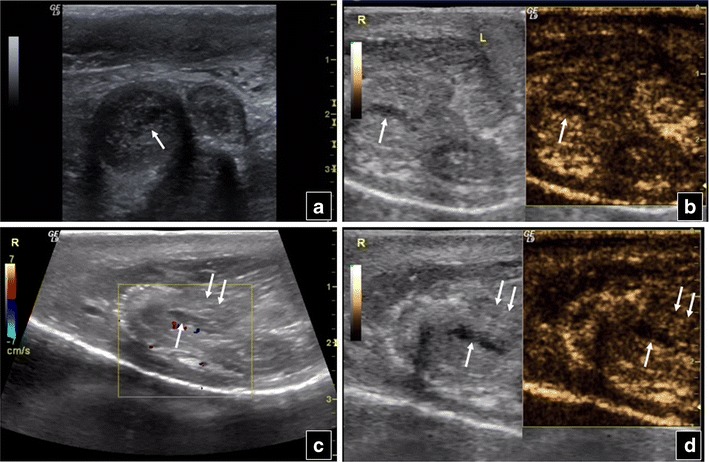
Transverse B-mode scan of the penis of a 36-year-old man (**a**) shows enlargement of the right corpus cavernosum penis, as well as an indistinct hypoechoic area (*arrow*) in its centre. This area shows no contrast enhancement on CEUS (*arrow* in **b**) and is consistent with an injury. Sagittal colour Doppler scan (**c**) also shows this traumatic area (*arrow*), while loss of surrounding fibrous tissue intactness (*double arrows*) is suggested. The findings of corpus cavernosum injury (*arrow*) and surrounding tissue rupture (*double arrows*) are confirmed in the sagittal CEUS view (**d**)

Baseline US may also suggest penile trauma, showing enlargement and inhomogeneous echogenicity of the corpora cavernosa penis or urethrae, focal contusions and loss of surrounding fibrous tissue intactness. Injuries appear as enhancement defects post contrast injection, elucidating or confirming conventional US findings (Fig. 12).

#### Segmental testicular infarction

Infarction appears on baseline US as a wedge-shaped area with decreased or absent colour flow [50]. CEUS helps in differentiating segmental rounded infarcts from poorly vascularised tumours, improving characterisation of a lesion as an infarct by detecting ischaemic parenchymal lobules separated by normal testicle vessels [51]. Subacute segmental infarctions show a peripheral enhancing rim, gradually diminishing and finally vanishing with changes in lesion shape and shrinkage [34].

#### Testicular infection

Acute epididymo-orchitis usually begins in the epididymal head or tail [43, 52], then spreading to the rest of the epididymis and the testicle. Sonography reveals an enlarged, hypoechoic or heterogeneous epididymis and testicle with increased blood flow. This finding has sensitivity as high as 100 % [53]. Focal orchitis produces hypoechoic areas with rich vascularity. Oedema, venous infarction and haemorrhage are sonographically similar to torsion [52]. Occasionally, untreated patients may be complicated with an abscess, where CEUS outlines peripheral uptake, no enhancement in the central liquefied content [44] and enhancing internal septations possibly missed on baseline US [54]. It can also differentiate non-enhancing haematomas from tumours and detect spermatic cord vessels thrombosis, especially in funiculitis [34].

### CEUS for urgent examination of vessels

As US contrast agents remain in the vascular lumen, they offer imaging comparable to that of angiography, without nephrotoxic iodinated contrast media and ionising radiation. In most cases, CEUS confirms findings of colour Doppler US or overcomes its limitations (breathing or heart motion artefacts, slow flow, flow in critical stenoses). High- and low-velocity flow phenomena are registered with CEUS without aliasing and blooming artefacts or angle dependence, factors compromising colour Doppler US examinations [55, 56]. US contrast agents can be used for practically all blood vessels of the body. Our department’s experience includes imaging of the abdominal aorta (AA), the inferior vena cava (IVC), the carotid arteries and the extremities vessels.

#### Large abdominal vessels indications

CEUS can improve imaging of AA aneurysms (AAA), by better delineating the lumen and main branches of the aorta [34]. In cases of suspected aneurysm rupture, it can image possible bleeding inside the thrombus, contrary to an intact thrombus which does not enhance. Colour Doppler occasionally produces false flow inside the aneurysmal thrombus due to motion artefacts. Absent CEUS enhancement rules out intrathrombus haemorrhage. On the contrary, in cases with intrathrombus leaks, contrast flows from the lumen towards the thrombus, while aortic rupture is seen as contrast extravasation and retroperitoneal haemorrhage [36, 57]. If the blood quantity is large, a pulsatile exit of contrast can be seen. A small quantity shows continuous flow [36, 57, 58]. CEUS also improves differentiation of ruptured aneurysms (which enhance) from pseudoaneurysms with no ongoing haemorrhage (which do not enhance) and can aid therapeutic treatment by guiding thrombin injection during leakage repair [58].

Abdominal aorta dissection usually happens as an extension of thoracic aortic dissection [57]. Up to 38 % of dissections are missed on initial US and up to 28 % remain undetected until autopsy [59–61]. CEUS can detect enhancement in both true (earlier) and false (later) lumen, if no thrombosis is present in the false lumen [60] and image complications, such as renal or splenic infarction [55]. However, CEUS has not yet been established routinely for urgent aortic imaging and its role is still questionable.

**Fig. 13:**
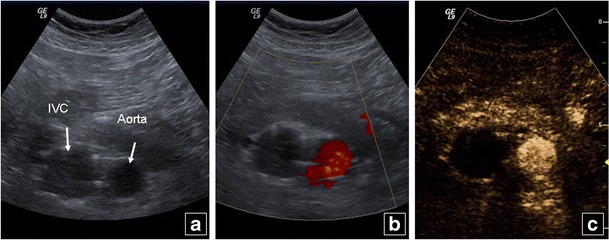
A 74-year-old male patient with bilateral leg deep venous thrombosis history and IVC filter. B mode US (**a**) shows a dilated IVC, while no blood flow is noted on colour Doppler (**b**). IVC thrombosis is confirmed on CEUS (**c**) with absence of contrast in the IVC, contrary to the normally enhancing abdominal aorta

Urgent diagnosis of previously unknown IVC thrombus is also a frequent indication for CEUS, which can better delineate the lumen and avoid motion artefacts, confirming complete or partial thrombosis suggested on colour and power Doppler US (Fig. 13).

A complication of AAAs with rupture inside the IVC, aortocaval fistula, can also be imaged with CEUS. The “gold standard” imaging method is CT angiography, with MR angiography (MRA) as an alternative. CEUS can be helpful in haemodynamically unstable patients, imaging blood flowing from aorta to IVC [62].

#### Carotid arteries

**Fig. 14:**
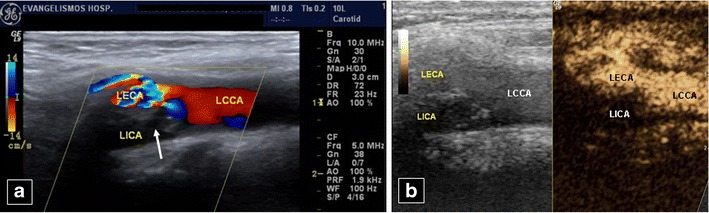
A 55-year-old woman. No flow is seen in the left internal carotid artery (*LICA*) on colour Doppler US (**a**), obviously due to a mixed echogenicity plaque (*arrow*) at its origin. The left common (*LCCA*) and external (*LECA*) carotid arteries and branch are patent. Occlusion of the LICA is confirmed post contrast injection (**b**). LCCA, LECA and branch enhance normally, while no contrast is present in the LICA. In this case CEUS confirmed colour Doppler findings, increasing confidence of the performing radiologist at 3:00 in the morning, without the need for an urgent angiography

Urgent carotid CEUS can confirm obstruction or dissection suggested on baseline colour US, overcoming its limitations, improving sensitivity [34] and confidence instantly, without the delay or radiation of angiography (Fig. 14). It can differentiate occlusion from tight sub-occlusive stenosis, improving delineation of endovascular border in difficult cases. Thus, CEUS allows detection of pre-stenotic, intra-stenotic and post-stenotic segments, especially in elongated vessels [55, 56]. Furthermore, it evaluates re-stenosis post internal carotid artery stenting. Since it presents fewer intrastenotic flow artefacts compared with colour Doppler, it better shows complete stenotic length and morphology [63].

**Fig. 15:**
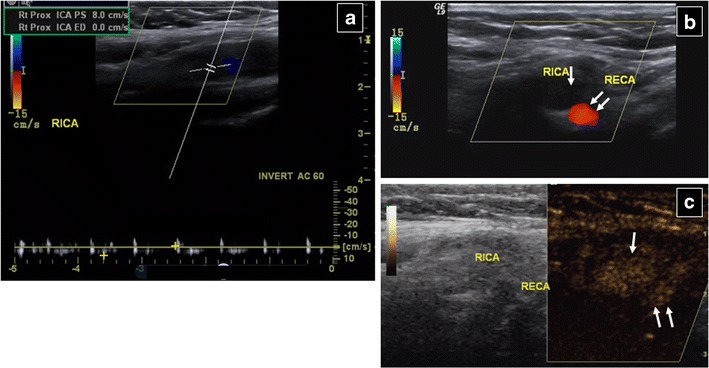
A 50-year-old woman with loss of consciousness. No measurable blood flow can be seen in the right internal carotid artery (*RICA*) on spectral (**a**) and colour (**b**) Doppler US. The external carotid (*RECA*) is patent. However, on CEUS (**c**) blood flow can be seen both in the RICA (*arrow*) and RECA (*double arrow*). Angiography performed later (not shown) detected more cephalad RICA dissection. In this case CEUS revealed poor, but present, blood flow in the RICA, which was not able to be detected by colour and spectral Doppler US, which suggested more caudal obstruction

Another carotid CEUS indication is dissection. Although magnetic resonance imaging (MRI) is the reference standard, before its performance or upon contraindications, CEUS can increase diagnostic accuracy of baseline US [55, 56], by revealing decreased but present blood flow (contrary to absent flow due to obstruction), a finding suggesting more cephalad occlusion that may be unclear on colour Doppler US (Fig. 15). The dissection flap may also be imaged.

#### Peripheral vessels

**Fig. 16:**
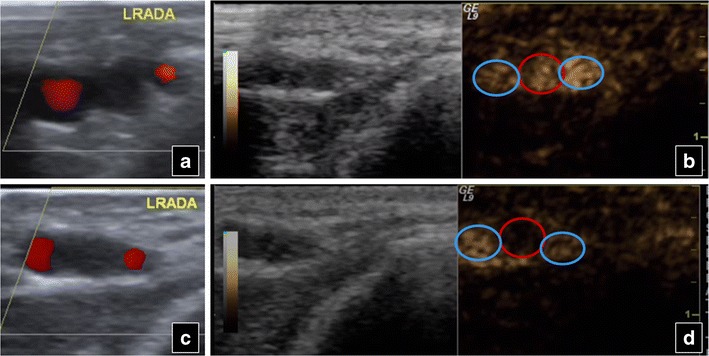
A 27-year-old man, an intravenous drug user, presents with left arm pain. Colour Doppler US shows an obstructed radial artery both in the upper (**a**) and in the lower (**c**) part of the antebrachium. The accompanying veins are patent at both levels. CEUS images a patent radial artery in the upper (*red circle* in **b**) but an obstruction (*red circle* in **d**) in the lower part of the antebrachium. The veins (*blue circles* in **b** and **d**) are patent. In this case CEUS overcame colour Doppler limitations and detected the level of occlusion

Extremity vessels can be even easier to examine with CEUS than abdominal vessels, since they are superficially located and not obscured by overlying tissues. Thrombosis, embolism, occlusion (Fig. 16), pseudoaneurysm, etc., suggested on baseline US, can be confirmed.

## Limitations of urgency CEUS

Urgency CEUS has not reached high availability worldwide and is strongly operator dependent [64], while highest value equipment and special software are needed. Additional time is required to prepare and administer the contrast agent, as well as to place an intravenous catheter. The high drug additional cost can be reduced by using only the needed dose for different indications, with the rest administered to another patient. Furthermore, CEUS cannot overcome problems related to lesion location (pancreas behind overlying bowel or gastric gas, fatty liver, aorta in obese patients, carotid artery with extensive wall calcification or post intervention subcutaneous emphysema [34]).

Finally, injection should be avoided as a precaution in patients with serious cardiopulmonary disease [17–19]. However, these patients are fewer than those with CT-MR contraindications due to anaphylactic history or impaired kidney function.

## Conclusion

Contrast-enhanced ultrasound is widely indicated for assessing urgency patients. It should always be performed after a baseline, non-enhanced scan if this is not diagnostic or to increase confidence in its findings. The method is easy to learn and fast to perform. In experienced hands, urgent CEUS can solve a wide variety of diagnostic problems.
